# Supplementary material for: Chitosan Catalyzed Novel Piperidinium Dicoumarol: Green Synthesis, X-ray Diffraction, Hirshfeld Surface and DFT Studies
Source: Polymers (Basel). 2022 Apr 30;14(9):1854. doi: 10.3390/polym14091854 (PMC9105048; doi:10.3390/polym14091854)
Supplement: Supplementary file 1 [file polymers-14-01854-s001.zip › polymers-1697811-SI.pdf]

polymers

MDPI

Supplementary Material: Chitosan Catalyzed Novel Piperidinium Dicoumarol: Green Synthesis, X-ray Diffraction, Hirshfeld Surface and DFT Studies

Mohammad Asad, Muhammad Nadeem Arshad, Mohammed Musthafa T. N. and Abdullah M. Asiri

Table S1. Bond Lengths for **4.**

Atom	Atom	Length/Å	Atom	Atom	Length/Å
C1	C21	1.514(3)	C15	O2	1.376(3)
C1	C3	1.523(3)	C15	C16	1.378(3)
C1	C12	1.524(3)	C16	C17	1.362(4)
C2	O4	1.215(2)	C17	C18	1.380(4)
C2	O1	1.385(2)	C18	C19	1.378(3)
C2	C3	1.439(3)	C20	C21	1.335(3)
C3	C4	1.379(3)	C20	O3	1.359(2)
C4	O8	1.301(2)	C21	C22	1.458(3)
C4	C5	1.471(3)	C22	O6	1.232(2)
C5	C10	1.383(3)	C22	C23	1.464(3)
C5	C6	1.386(3)	C23	C24	1.378(3)
C6	O1	1.365(2)	C23	C28	1.401(3)
C6	C7	1.400(3)	C24	O3	1.373(2)
C7	C8	1.375(3)	C24	C25	1.387(3)
C8	C9	1.375(3)	C25	C26	1.366(3)
C9	C10	1.370(3)	C26	C27	1.383(4)
C11	O5	1.216(3)	C27	C28	1.368(3)
C11	O2	1.372(2)	C29	N1	1.480(5)
C11	C12	1.434(3)	C29	C30	1.487(4)
C12	C13	1.361(3)	C30	C31	1.504(5)
C13	O7	1.318(2)	C31	C32	1.501(4)
C13	C14	1.460(3)	C32	C33	1.487(4)
C14	C15	1.368(3)	C33	N1	1.456(4)
C14	C19	1.389(3)			

Table S2. Bond Angles for **4.**

			4				
**C21**	**C1**	**C3**	**117.43(16)**	**C14**	**C15**	**O2**	121.26(18)
C21	C1	C12	110.63(15)	C14	C15	C16	122.0(2)
C3	C1	C12	114.74(15)	O2	C15	C16	116.7(2)
O4	C2	O1	113.70(18)	C17	C16	C15	119.1(3)
O4	C2	C3	127.1(2)	C16	C17	C18	120.5(2)
O1	C2	C3	119.22(19)	C19	C18	C17	119.9(3)
C4	C3	C2	120.27(19)	C18	C19	C14	120.2(3)
C4	C3	C1	124.83(18)	C21	C20	O3	125.71(19)
C2	C3	C1	114.82(17)	C20	C21	C22	118.77(18)
O8	C4	C3	123.87(19)	C20	C21	C1	124.49(18)
O8	C4	C5	117.18(18)	C22	C21	C1	116.65(17)
C3	C4	C5	118.95(18)	O6	C22	C21	122.55(19)
C10	C5	C6	118.10(19)	O6	C22	C23	122.00(19)

C10	C5	C4	123.63(19)	C21	C22	C23	115.44(18)
C6	C5	C4	118.24(19)	C24	C23	C28	117.8(2)
O1	C6	C5	122.00(19)	C24	C23	C22	120.38(19)
O1	C6	C7	116.1(2)	C28	C23	C22	121.9(2)
C5	C6	C7	121.9(2)	O3	C24	C23	121.38(19)
C8	C7	C6	117.8(2)	O3	C24	C25	116.3(2)
C7	C8	C9	121.0(2)	C23	C24	C25	122.4(2)
C10	C9	C8	120.4(2)	C26	C25	C24	118.3(2)
C9	C10	C5	120.7(2)	C25	C26	C27	120.9(2)
O5	C11	O2	115.34(19)	C28	C27	C26	120.2(2)
O5	C11	C12	125.59(19)	C27	C28	C23	120.4(2)
O2	C11	C12	119.1(2)	N1	C29	C30	111.8(3)
C13	C12	C11	119.70(18)	C29	C30	C31	111.2(3)
C13	C12	C1	125.19(18)	C32	C31	C30	109.6(3)
C11	C12	C1	114.92(18)	C33	C32	C31	112.2(3)
O7	C13	C12	124.07(17)	N1	C33	C32	111.2(3)
O7	C13	C14	115.80(19)	C33	N1	C29	112.4(2)
C12	C13	C14	120.13(19)	C6	O1	C2	121.13(17)
C15	C14	C19	118.3(2)	C11	O2	C15	121.56(18)
C15	C14	C13	118.2(2)	C20	O3	C24	118.25(16)
C19	C14	C13	123.5(2)				

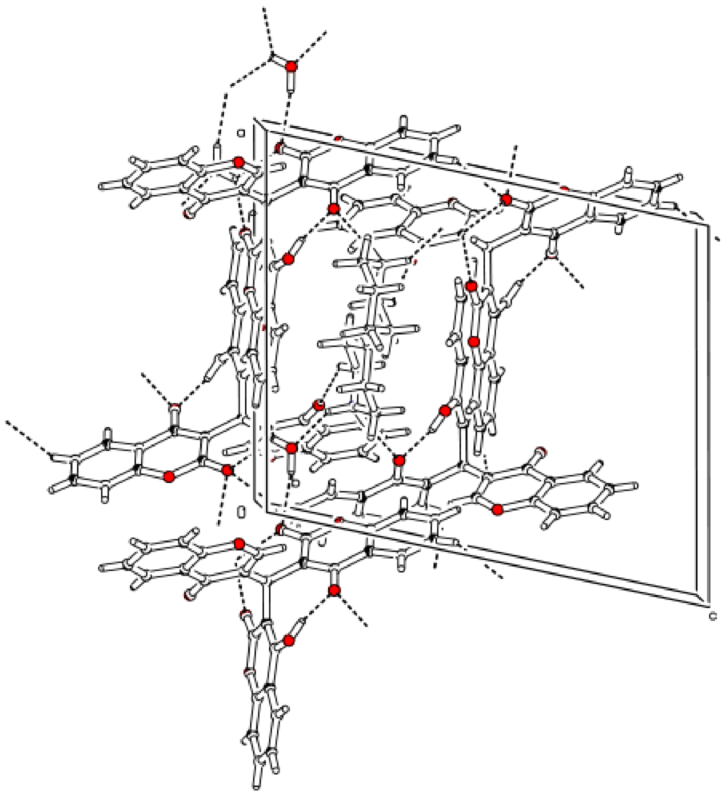

Figure S1. A unit cell diagram showing the hydrogen bonding interactions.

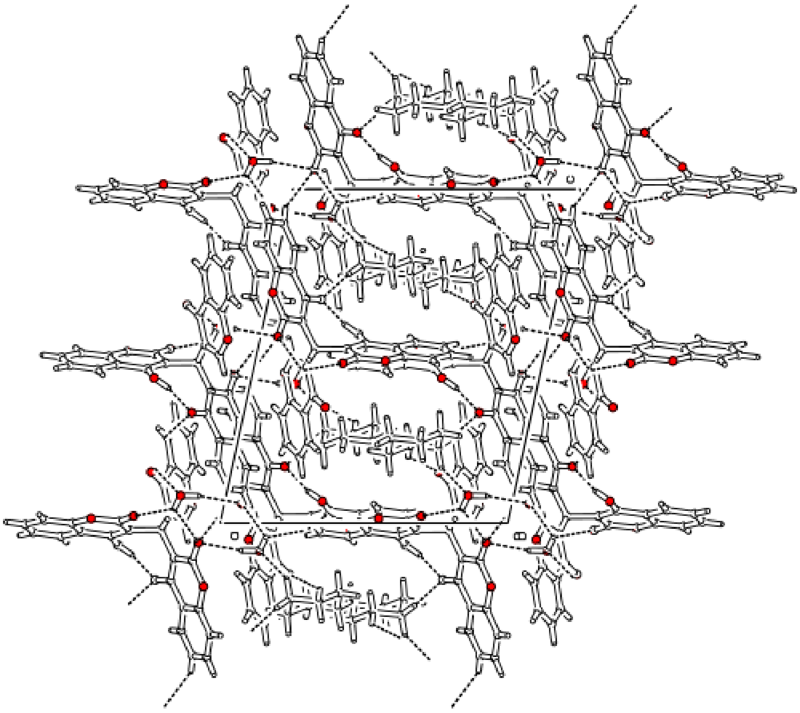

Figure S2. Packing diagram showing the formation of 3-dimensional network
